# Crystal structure of human aldehyde dehydrogenase 1A3 complexed with NAD^+^ and retinoic acid

**DOI:** 10.1038/srep35710

**Published:** 2016-10-19

**Authors:** Andrea Moretti, Jianfeng Li, Stefano Donini, Robert W. Sobol, Menico Rizzi, Silvia Garavaglia

**Affiliations:** 1 Department of Pharmaceutical Sciences, University of Piemonte Orientale, Largo Donegani 2, 28100 Novara, Italy; 2 Department of Oncologic Sciences, Mitchell Cancer Institute, University of South Alabama, Mobile, AL 36604, USA

## Abstract

The aldehyde dehydrogenase family 1 member A3 (ALDH1A3) catalyzes the oxidation of retinal to the pleiotropic factor retinoic acid using NAD^+^. The level of ALDHs enzymatic activity has been used as a cancer stem cell marker and seems to correlate with tumour aggressiveness. Elevated ALDH1A3 expression in mesenchymal glioma stem cells highlights the potential of this isozyme as a prognosis marker and drug target. Here we report the first crystal structure of human ALDH1A3 complexed with NAD^+^ and the product all-*trans* retinoic acid (REA). The tetrameric ALDH1A3 folds into a three domain-based architecture highly conserved along the ALDHs family. The structural analysis revealed two different and coupled conformations for NAD^+^ and REA that we propose to represent two snapshots along the catalytic cycle. Indeed, the isoprenic moiety of REA points either toward the active site cysteine, or moves away adopting the product release conformation. Although ALDH1A3 shares high sequence identity with other members of the ALDH1A family, our structural analysis revealed few peculiar residues in the 1A3 isozyme active site. Our data provide information into the ALDH1As catalytic process and can be used for the structure-based design of selective inhibitors of potential medical interest.

Living organisms are constantly confronted with oxidative stress and with the reactive oxygen species (ROS) derived therefrom. In animals, a plethora of processes contribute to ROS formation and the superfamily of aldehyde dehydrogenase enzymes (ALDHs) are known to decrease oxidative stress, particularly that caused by aldehydes. During numerous physiological processes, aldehydes are generated from a variety of precursors, including the endogenous biotransformation of most compounds such as amino acids, neurotransmitters, carbohydrates and lipids^1^,^2^. While some aldehydes play vital roles in normal physiological processes like vision, embryonic development and neurotransmission, many others are cytotoxic and carcinogenic^3^. The ALDHs superfamily consists of 19 putatively functional members that are involved in the clearance of potentially toxic aldehydes and play essential roles for the production of key metabolic regulators^2^,^4^. Most of the ALDHs have wide tissue distribution and display distinct substrate specificities^2^,^5^. The ALDH enzymes catalyse the NAD(P)^+^-dependent irreversible oxidation of a wide spectrum of endogenous and exogenous aldehydes and are found in all subcellular regions including the cytosol, mitochondria, endoplasmic reticulum and nucleus, often being expressed in more than one compartment^6^.

In particular, three members of the cytosolic aldehyde dehydrogenase class 1 (ALDH1A1, ALDH1A2, ALDH1A3) have a key role in vertebrate development. Indeed, they show a high specificity for retinaldehyde that they oxidise to the potent differentiation factor retinoic acid (REA)^7^. The retinoic-acid signalling pathway in vertebrates utilizes two classes of retinoid receptors, RARs and RXRs that belong to the family of nuclear hormone receptors. These proteins are ligand-regulated transcription factors that bind 9-*cis* (RXR, RAR) or all-*trans* (RAR) retinoic acid via a ligand-binding domain, and direct the transcription of target genes via a DNA-binding domain^8^,^9^. Retinoic acid is derived from vitamin A (retinol) and its pleiotropic effects include spinal chord and retina development during embryogenesis, neuronal cell differentiation and maintenance of epithelial cell type in adult tissues^10^. Synthesis of REA proceeds through two steps: the oxidation of retinol by alcohol dehydrogenase producing the relative aldehyde, followed by its irreversible conversion to acid using retinal dehydrogenases^11^. The ALDH1A3 isozyme shares more than 70% sequence identity with ALDH1A1 and ALDH1A2 that includes residues involved in catalysis. Although ALDH1A1, 1A2 and 1A3 recognize a common substrate, their expression pattern does not overlap entirely and reflects their preference for specific substrates. Indeed, ALDH1A1 preferentially processes aldehydes produced by lipid peroxidation while retinal is the preferred substrate for both ALDH1A2 and ALDH1A3^12^. In addition, murine ALDH1A3 processes *all*-trans retinal with a catalytic efficiency that is 10-fold higher than that of ALDH1A1 and ALDH1A2^13^. ALDH1A3 is differentially activated during early embryonic head and forebrain development, and it is expressed at high levels in the differentiating keratinocytes of human and murine hair shafts^14^,^15^. Moreover, knockout of the murine Aldh1a3 gene was associated with perinatal lethality that could be rescued by maternal treatment with REA^16^.

The milieu of cancerous tumours consists of heterogeneous cell populations, and increasing evidence indicates that cancer stem cells (CSCs) have the potential for self-renewal and differentiation able to drive tumourigenesis^17^. In this context, high ALDHs activity is being considered as a potential prognostic marker for cancer since its increase in CSCs correlates with a poor outcome for many solid tumours^18^,^19^. Moreover, compelling evidence highlight ALDHs as key molecules governing cell proliferation, survival and chemoresistance of CSCs^20^ and strongly suggests that the development of potent and selective inhibitors may represent a novel CSC-directed therapeutic potential in human cancers^21^,^22^. In this respect, it should be noted that distinct isozymes have a unique relevance in different tumours; for instance, ALDH1A1 is proposed as a sensitive target in human melanoma^18^ whereas ALDH1A3 is considered a target in breast cancer^22^. Moreover, high expression of ALDH1A3 has recently been observed in clinical high-grade gliomas (HGGs) but not in low-grade gliomas or in normal brain samples^23^. Two distinct tumour-derived GSC subtypes were identified in high-grade glioma: the proneuronal glioma stem cells (PN GSCs) and the mesenchymal glioma stem cells (Mes GSCs) with elevated expression of human ALDH1A3 being observed in Mes GSCs with respect to PN GSCs^23^. HGGs generated from Mes GSCs display a significantly higher radio-resistance and irradiation has been shown to induce ALDH1A3 up-regulation that is involved in the PN GSCs transformation into Mes GSCs^23^. Taken together, these data suggest that human ALDH1A3 may represent a valuable target for the development of novel therapeutics against HGGs^23^.

Here we report on the first crystal structure of the human ALDH1A3 complexed with REA and NAD^+^, which has been determined at 2.9 Å resolution. The structure determination of ALDH1A3 in complex with ligands provides novel information to understand catalysis in the ALDH1As subfamily and represents a valuable tool for the structure-based design of potent and selective inhibitors of medical interest as new anticancer agents.

## Results and Discussion

### Biochemical characterization

The ALDH1A3 kinetic parameters were determined for NAD^+^, the natural substrate all-*trans* retinal and acetaldehyde, a general substrate accepted by many ALDH isozymes^24^. All the compounds tested showed Michaelis–Menten kinetics with parameters reported in Supplementary Table S1. The steady state kinetic data are consistent with those reported for others ALDH1s^25^,^26^. In particular, the observed 258-fold decrease in the K_M_ value for all-*trans* retinal (K_M_ = 9.3 × 10^−6^ M) with respect to acetaldehyde (K_M _= 2.4 × 10^−3^ M), confirms the substrate specificity of human ALDH1A3 for retinal. Moreover, as reported for other dehydrogenases the measured K_M_ for NAD^+^ is almost 2 times lower than the K_M_ for retinal^27^. Noteworthy, the human ALDH1A3 turnover number for the all-*trans* retinal oxidation (k_cat_ = 1.6 s^−1^) is about 12 and 18 times higher that those reported for mouse ALDH1A2 and human ALDH1A1, respectively^25^ confirming 1A3 as the isozyme with the higher enzymatic activity for this substrate. The thermal stability of ALDH1A3 in complex with NAD^+^, NADH, all-*trans* retinal and all-*trans* retinoic acid (REA) was investigated by a thermal shift assay. As reported in Supplementary Table S2, only NAD^+^ significantly stabilizes the enzyme. This is in agreement with a previous report that demonstrate, the sequential ordered mechanism of the ALDHs, in which the cofactor is the first substrate to bind to the enzyme active site^28^.

### Overall quality of the model

The three-dimensional structure of human ALDH1A3 has been solved by molecular replacement using the atomic coordinates of ALDH1 sheep liver as a search model (PDB code: 1BXS)^29^. The final human ALDH1A3 model contains eight identical chains per asymmetric unit, arranged as two independent tetramers and a total of 63 solvent molecules. In each monomer no electron density is present for the N-terminal portion and for the last four amino acids at the C-terminus. In addition, no electron density was observed in specific regions in different monomers as detailed in Supplementary Table S3. The height monomers present in the asymmetric unit do not display any significant conformational difference and show an average r.m.s. deviation of 0.44 Å after optimal superposition based on all 475 Cα atoms. Amongst the two independent tetramers present in the asymmetric unit, the ABCD tetramer shows the lowest degree of disorder (Supplementary Table S3) and is therefore used as a reference from hereon as well as the monomers C and D when referring to a dimer. Each monomer binds one molecule of NAD^+^ and one molecule of the product REA. The stereochemistry and the structure geometry of the final model has been assessed with the program RAMPAGE^30^ and shows 99% of protein residues in the favoured regions of the Ramachandran plot.

### Overall structure of human ALDH1A3

#### The tetramer

**Figure 1:**
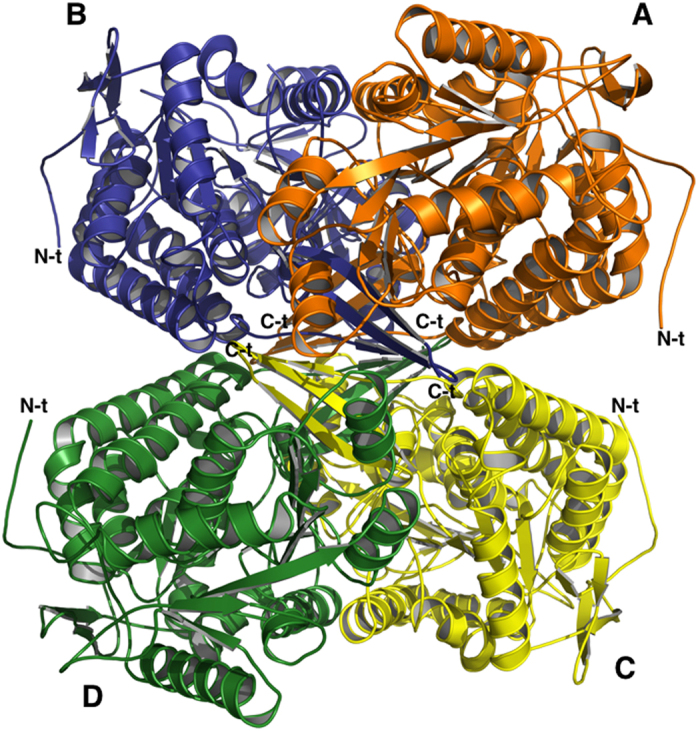
The oligomeric structure of human ALDH1A3. Ribbon representation of the hALDH1A3 tetramer with chains (**A–D**) coloured in orange, blue, yellow and green, respectively. The N-terminals are highly mobile and exposed on the surface of the tetramer while the C-terminals point toward its core. N-t →N-terminus; C-t →C-terminus.

ALDH1A3 has a predicted molecular weight of 56108 Da and our gel filtration experiment shows a native molecular mass of about 224000 Da corresponding to one tetramer (data not shown), as already described for others ALDHs^31^. The crystal structure of ALDH1A3 reveals a tetrameric assembly with intimately associated monomers (Fig. 1). Indeed, the analysis carried out with the software PISA^32^ calculated an Assembled Surface Area (ASA) of 62658,6 Å^2^ and a Buried Surface Area of 17769,6 Å^2^ for the tetramer. In particular, within the tetramer, the most extensive contact surface is observed between monomers A and B (BSA 2520 Å^2^) and C and D (BSA 2456 Å^2^). The major contacts at the dimer interface are provided by the β5, α7, β18, β15 and α13 secondary structures and mainly consist of hydrogen bonds with few hydrophobic interactions. Three salt bridges between R84→E426, R487→E489 and R271→G277 were also identified that favour the dimers stability.

#### The monomer

**Figure 2:**
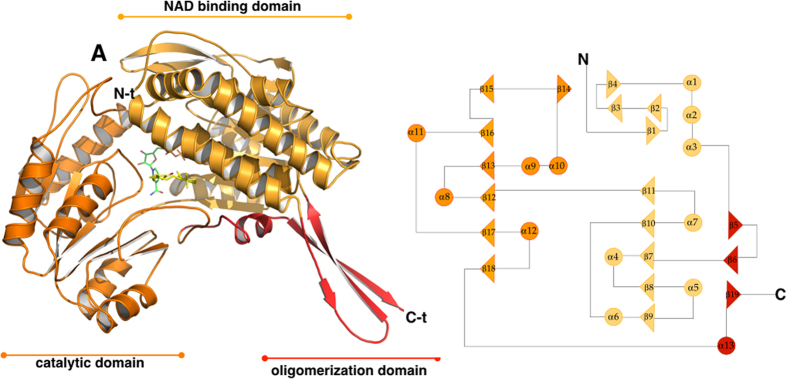
The overall structure of human ALDH1A3 monomer. Left hand side: ribbon representation of the hALDH1A3 monomer with its three domains coloured differently: the NAD binding-domain is shown in light orange, the catalytic-domain in orange and the oligomerization domain in red. The ligands NAD^+^ and REA are shown as green and yellow sticks, respectively. Right hand side: topology diagram of hALDH1A3.

Despite human ALDH1A3 assembling as a tetramer, each monomer is independently able to convert one molecule of substrate to one molecule of product by reducing one NAD^+^. The structure analysis confirms that ALDH1A3 shares high structural homology with all classes of ALDHs and folds into 13 α-helices, 19 β-strands and the connecting loops, arranged into three functional domains (Fig. 2). The N-terminal NAD binding domain (L20-D149 and I171–G282) contains a five-stranded parallel β-sheet (β9, β8, β7, β10 and β11) while the catalytic domain (G283–M482) is featured by a six-stranded parallel β-sheet (β15, β16, β13, β12, β17 and β18) and the oligomerization domain (K150–P170 and S483–L507) by a three-stranded antiparallel β-sheet (β5, β6 and β15). Both the NAD^+^ binding domain and the catalytic domain are built on a topologically related βαβ type polypeptide fold (Fig. 2). As expected, ALDH1A3 shows strong conservation with other members of the 1A subfamily. Indeed, the 1A3 structure can be optimally superimposed onto human ALDH1A1 (PDB: 4WB9)^33^ with an r.m.s.d. of 0.84 Å based on 485 equivalent Cα pairs, and shares 71% amino acid identity with human isozymes 1A1 and 72% amino acid identity with 1A2. However, as detailed in the following sections, a few single unique amino acids can be observed within the ligand-binding pocket of the different isoforms.

### REA and NAD^+^ display different and coupled conformations

**Figure 3:**
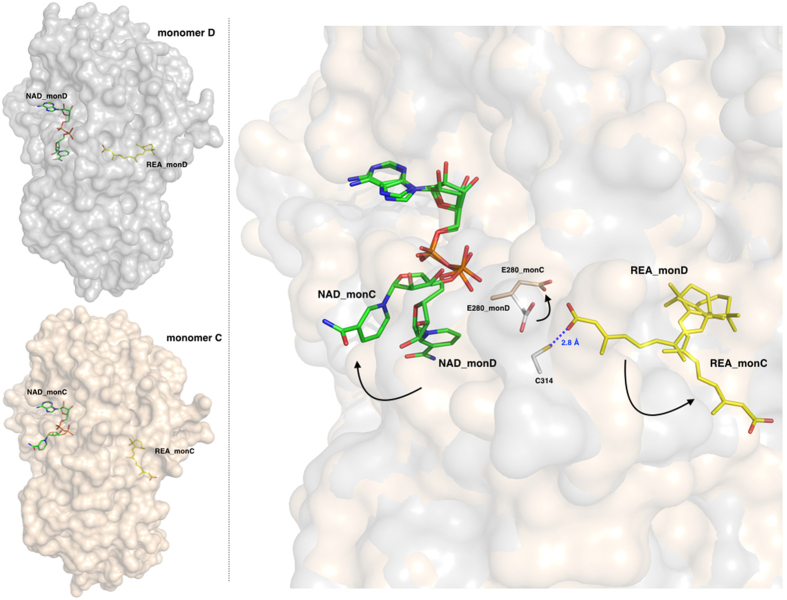
The two different and coupled conformations adopted by REA and NAD^+^. Left-hand side: surface representation of monomers D (upper, in grey) and C (lower, in light-pink) with bound NAD^+^ and REA shown as green and yellow sticks, respectively. Right-hand side: a zoom-in showing the REA and NAD^+^ conformations as observed in monomers D and C, shown with a surface representation in the background, after optimal superposition; the catalytic C314 from monomer D is depicted as grey stick and the E280 is shown in the two conformations adopted in monomer D and C. The two conformations observed for REA and NAD^+^ are coupled as indicated by the arrows, one couple representing the closed state (in monomer D) and the open state (in monomer C) that represents the product-release conformation. The residue E280 also oscillates between two conformations following those observed for the ligands.

Our crystallographic data revealed that both REA and NAD^+^ bind to the enzyme active site with two different conformations that we observed in different monomers. In particular, in the monomers A and D, REA displays a closed conformation with its carboxyl group pointing toward the catalytic residue C314. In the same monomers, the nicotinic acid moiety of NAD^+^ is oriented in the direction of the active site. On the other hand, in monomers B and C, the REA adopts what we propose to be the product conformation, with its isoprenic chain moving away from the active site toward the protein surface, so that the entire REA molecule efficiently occupies a tunnel that provides substrate access to and product release from, the catalytic cysteine. In parallel, the NAD^+^ nicotinic ring also moves away from the active site pointing toward the protein surface (Fig. 3). In summary, we observed two different and linked conformations for the REA-NAD^+^ couple in two different monomers: the first showing the REA carboxylate pointing toward the C314 and the second showing the product conformation with the REA carboxylate moiety oriented toward the protein surface. The NAD^+^ and REA coupled conformations do not appear to be determined or influenced by contacts occurring between different monomers within the tetrameric assembly or from crystallographic symmetry mates. Therefore, we can surmise that in human ALDH1A3 structure, the different conformations adopted by the ligands are the consequence of specific interactions with ALDHs conserved residues.

#### The REA binding site

**Figure 4:**
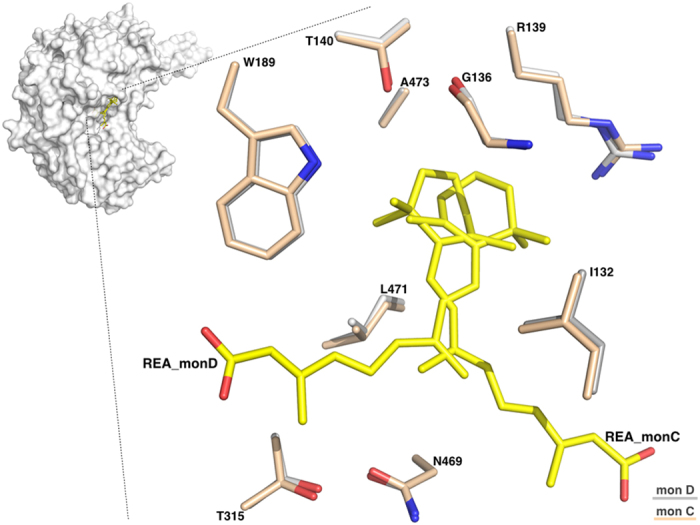
The REA binding site. Zoom-in of the REA binding site showing the two conformations adopted by REA in monomers D and C, after optimal superposition. Residues from the two monomers are represented as sticks, and coloured in light-pink for monomer C and in grey for monomer D. The two REA molecules (REA_C and REA_D) are represented as sticks and shown in yellow.

**Figure 5:**
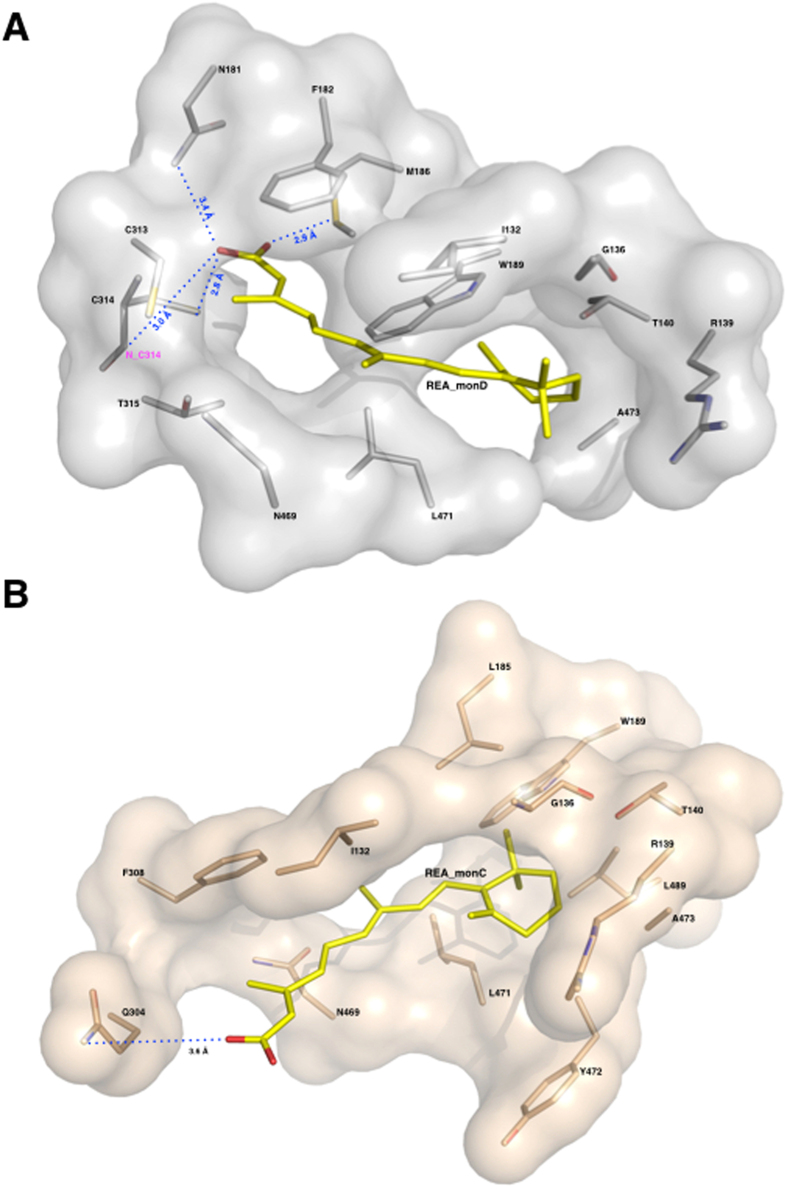
The REA binding site and major interactions established by REA with the protein milieu in its two conformations. Surface representation of REA binding sites: the monomers D in grey and the monomer C in light-pink. Protein residues are represented as sticks and coloured in light-pink for monomer C and in grey for monomer D; the REA ligands (REA_C and REA_D) are shown as sticks and depicted in yellow. (**A**) The REA binding mode as observed in monomer D. The β-ionone ring establishes hydrophobic interactions with I132, G136, R139, T140, W189, L471 and A473. Its carboxyl group makes hydrogen bonds with C314, M186 and N181, and hydrophobic interactions with F182, C313, T315 and N469. (**B**) The REA binding mode as observed in monomer C. The β-ionone ring maintains the same interactions as in monomer D. On the contrary its carboxyl group significantly moves and makes hydrogen bonds with Q304 and hydrophobic interactions with F305 and N469.

An important feature revealed by our structural studies is the precise definition of the catalytic pocket that consists of a tunnel connecting the protein surface to the catalytic cysteine, and that likely also represents the pathway for substrate access and product release, as previously predicted for sheep liver and human ALDH1A1^32^,^33^. In human ALDH1A3 the catalytic pocket is mainly contributed by three α helices and a surface loop. Helix α3 defines the left-hand side of the opening and forms part of a three-helix bundle that characterizes the N-terminal domain of ALDH1s. The right-hand side of the tunnel is contributed by the surface loop (467–473) that precedes the α13 helix part of the oligomerization domain. The back of the tunnel is delimitated by helix α4, the first α-helix of the canonical Rossmann fold present in the N-terminal domain, and is also contributed by two β-strands (β10, β11). Finally, helix α8, that belongs to the catalytic domain and immediately precedes the active-site nucleophile C314, makes up the bottom of the tunnel (Fig. 2). The major mechanism adopted by ALDHs to govern substrate specificity relies on structural features of the substrate access tunnel that ends up at the catalytic cysteine. It has recently been proposed that three topologically conserved residues (positions 124, 459 and 303 with numbering referred to human mitochondrial ALDH2) that are located in the catalytic pocket, govern substrate specificity in different ALDHs^34^. In ALDH1A3 these positions are occupied by G136, L471 and T315. Our structural analysis revealed that G136 is located at the entrance of the tunnel and allows the admittance of large substrates while L471, positioned on the right-hand side of the tunnel, is a major determinant guiding selectivity in 1A3 and other members of the 1A subfamily by making hydrophobic contact with the REA isoprenic chain (3.74 Å) (Fig. 4). Finally, T315 that lies next to the reactive C314 also interacts with the substrate. In both conformations adopted by REA, its β-ionone ring does not stick out from the tunnel as previously speculated but instead sits at its entrance and establishes contacts with the protein environment, that involve a van der Waals contacts with I132, G136, R139, T140, W189, L471 and A473 (Fig. 4). As all these residues are conserved in both the 1A1 and 1A2 isozymes, such interactions are presumably stabilizing REA binding in all members of the 1A subfamily. As already mentioned above, REA binds to monomer D in a closed conformation, with its carboxyl group close to the active cysteine and extends its β-ionone and isoprenic chain moieties along the whole access-substrate tunnel. Indeed, the calculation of a 290 Å^2^ residual solvent-accessible area, highlights the efficient occupancy of the tunnel. The REA carboxyl group is located at the end of the tunnel and makes two stabilizing contacts with the strictly conserved M186 and C314. The thiol group of the catalytic C314 hydrogen bonds with the REA carboxylic O2 oxygen (2.8 Å) while the SD atom of M186 contacts the O1 oxygen (2.9 Å). Notably, the distance of the REA carboxyl carbon from the thiol group of the highly conserved catalytic cysteine (3.1 Å), is comparable with the one observed in the structure of hALDH2 in complex with the substrate crotonaldehyde (PDB:1O01) where the aldehyde carbonyl carbon is at 3.0 Å from the catalytic cysteine^35^. In addition the oxygen of the REA ε-carboxylate sits in the oxyanion hole that is built by the backbone N of the catalytic C314 and by the N2D of N181. The O1 of REA is 3.4 Å away from the N2D of the N181 and at 3.0 Å from the backbone N of the C314; this latter distance is compatible with the formation of a hydrogen bond as also reported for others ALDHs in complex with the product^29^,^36^ (Fig. 5B). The conformation observed for M186 significantly differs from that adopted in monomer C where REA moves away from the catalytic cysteine. In particular, M186 methyl group rotates by 45° to make room to the REA carboxylic moiety. In monomer C, REA binds in an open conformation with its isoprenic and carboxylic moieties pointing to the opening of the catalytic tunnel toward the protein surface. As a consequence, the calculated solvent-accessible area of the tunnel increases to a value of 310 Å^2^. The REA carboxyl group is partially exposed to solvent and stabilized by two interactions with protein residues. In particular, its O2 oxygen interacts with the H128 NE2 atom (5.6 Å) and O1 with the Q304 OE1 (3.6 Å) (Fig. 5B). A remarkable difference that we observe in the active site structure in monomers C and D regards the strictly conserved E280 that shows two different conformations. E280 is a key residue for catalysis in ALDHs and acts as the general base responsible of the activation of two water molecules required for the formation of the thiolate in the catalytic C314 and for the hydrolysis of the thioester intermediate. While in monomer D, E280 is in close contact with C314 at a distance of 3.4 Å, in monomer C it moves away from the catalytic cysteine and occupies an outer position (Fig. 3). As proposed by Muñoz-Clarez *et al.*^37^, such a structural rearrangement is required during catalysis and supports our view that the REA mode of binding observed in monomer D represents the substrate-like conformation.

As already detailed above, the three ALDH1 isozymes are highly structurally conserved. However, the structural superposition of the active sites of ALDH1A3 and ALDH1A1 surprisingly reveals two significant and specific amino-acid substitutions. In particular, we found that N469, located in the tunnel, is replaced by a glycine in the 1A1 isozyme and that T315, playing an important role in substrate selectivity, is replaced with an isoleucine in ALDH1A1s. Such a structural observation opens the way for the rational design of selective inhibitors targeting ALDH1A3.

#### The NAD^+^ binding site

**Figure 6:**
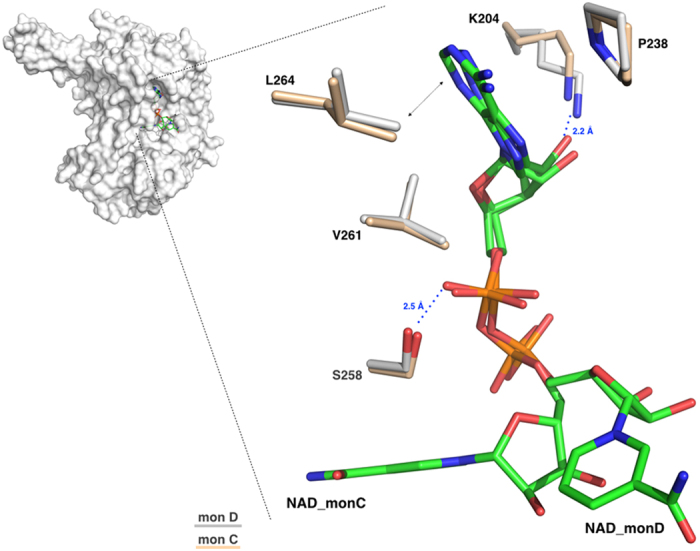
The NAD^+^ binding site. Zoom-in of the NAD^+^ binding site showing the two conformations adopted by the cofactor in monomers D and C, after optimal superposition. Residues from the two monomers are represented as sticks, and coloured in light-pink for monomer C and in grey for monomer D. The two NAD^+^ molecules (NAD_C and NAD_D) are represented as sticks and shown in green.

**Figure 7:**
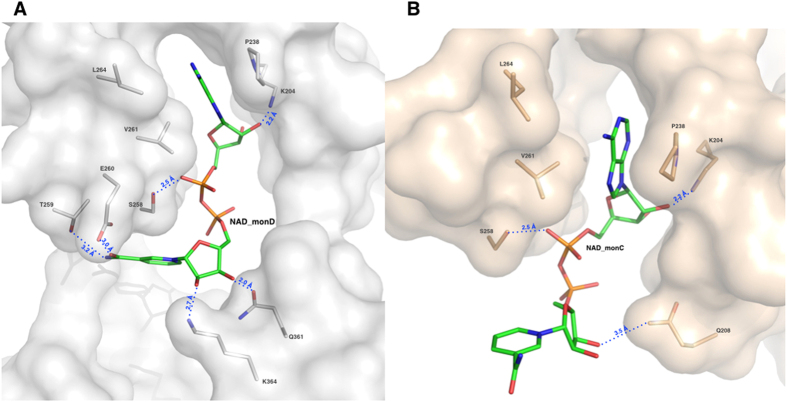
The NAD^+^ binding site and major interactions established by REA with the protein milieu in its two conformations. Surface representation of NAD^+^ binding sites: the monomers D in grey and the monomer C in light-pink. Protein residues are represented as sticks and coloured in light-pink for monomer C and in grey for monomer D; the NAD^+^ ligands (NAD_C and NAD_D) are shown as sticks and depicted in green. (**A**) The NAD^+^ binding mode as observed in monomer D with P238, L264 and V261 contacting the NAD^+^ adenine moiety and K204 and S258 the adenine ribose. The NAD^+^ nicotinamide moiety is stabilized through hydrogen bonds established with four amino-acids: K364, Q361, T259 and E260. (**B**) The NAD^+^ binding mode as observed in monomer C. The ADP moiety maintains same orientation as in monomer D and establishes same contacts with protein residues. On the contrary, the nicotinamide changes orientation and loses the network of interactions observed in its closed conformation in monomer D and the nicotinamide ribose makes a new contact with Q208.

The N-terminal NAD^+^-binding domain exhibits the typical ALDH cofactor-binding motif βαβ that differs from the classical Rossmann fold^38^. As observed in other human ALDH structures complexed with NAD^+^ (PDB code: 4WB9, 1O00, 1BXS), the dinucleotide adenine moiety lies in a conserved hydrophobic pocket located between helix α4 and strand β8 (Fig. 2). While the adenine, the adenine ribose and the two phosphate groups show the same conformation in all of the monomers, both the nicotinamide and the nicotinamide ribose are observed in two different conformations: open as observed in monomer C and closed as in monomer D. A number of stabilizing interactions sustain the NAD^+^ recognition indicating that the cofactor binds strongly to hALDH1A3 as also suggested by the increased enzyme thermal stability when complexed with NAD^+^ (Supplementary Table S2). In particular, the adenine base is held in place by van der Waals contacts with P238, V261 and L264; the adenine ribose by a hydrogen bond between its O2B and the K204 NZ atom (2.2 Å) and the first phosphate by another hydrogen bond made by its O1A oxygen with the OG of S258 (2.5 Å) (Fig. 6). The NAD^+^ nicotinamide moiety has been reported to be mobile and observed bound to different ALDHs with different conformations^39^. In this respect, also the structure of ALDH1A3 shows a significant mobility of the nicotinamide moiety. In the closed conformation, the nicotinamide group is oriented toward the catalytic site with a shorter distance of 8.3 Å from the Sγ of the catalytic cysteine. Indeed, the superposition with the NAD^+^ conformation observed in the sheep ALDH1A1 structure^29^, where the cofactor adopts the hydride transfer position, reveals that the C4 of the nicotinamide ring in the two structures are 5.4 Å apart. In this conformation, the nicotinamide ribose oxygens are stabilized by hydrogen bonds. In particular, the O2D contacts the NZ of K364 (2.7 Å) and the O3D engages the OE1 (2.0 Å) and NE2 (3.5 Å) of Q361. Finally, the carbamide group is also stabilized through hydrogen bonds established between its amide nitrogen and the OG1 of T259 (3.2 Å) and between its carbonyl oxygen and the OE1 of E260 (3.0 Å) (Fig. 7A). On the other hand, the open conformation positions the carbamide group 16.4 Å far away from the Sγ of catalytic cysteine. In this conformation, by moving close to the protein surface, the entire NAD^+^ nicotinamide moiety loses the network of interactions observed in the closed conformation. Moreover, also the nicotinamide ribose moves away from the catalytic cysteine and in the open conformation establishes only one hydrogen bond between its O3D atom and the NE2 of Q208 (3.5 Å) (Fig. 7B).

## Conclusion

Human ALDH1A3 has a key role in vertebrate development by participating in the synthesis of the differentiation factor retinoic acid^40^. In the present study we report the crystal structure of human ALDH1A3 complexed with NAD^+^ and the physiological product all*-trans* retinoic acid. Our structural investigation provides the first description of the REA binding site in a member of the ALDH1A subfamily. Both the REA and NAD^+^ shows two different conformations that appear to be coupled. Indeed, in the closed-in conformation, both ligands point the reactive like moieties of the molecule toward the active site cysteine. On the other hand, in the product-like conformation both NAD^+^ and REA move away from the catalytic pocket to the protein surface and occupy a binding pocket located at the entrance of the catalytic tunnel. Remarkably, the conformational changes adopted by the ligands concern the catalytic reactive moieties on both molecules. Indeed, it is the nicotinamide ring of NAD^+^ and the isoprenic chain of REA that move in a concerted manner either close to the active site cysteine or toward the protein surface, while both the REA β-ionone ring and the ADP moiety of NAD^+^ maintain the same conformation. Structural superposition with human ALDH1A1 reveals the expected high level of conservation; however, few significantly different residues are observed in structurally equivalent positions in the catalytic pocket. In particular, in ALDH1A3 an asparagine at position 469 and a threonine at position 315, respectively replace a glycine and an isoleucine present in ALDH1A1. Remarkably, both residues contact REA in the closed conformation and therefore play a role in ligand recognition. The last observation suggests that the structure-based design of potent and, most importantly, specific inhibitors of potential medical interest, is achievable.

## Methods

### Expression and purification of human ALDH1A3

The mesenchymal (Mes) Glioma stem cell (GSC) line 83 was a generous gift from I. Nakano to RWS^23^. This Mes GSC cell line was developed at the Ohio State University under an IRB-proved protocol according to NIH guidelines, as described^23^. Total RNA was isolated from the Mes GSC cell line 83 and was used to generate a Mes GSCs cDNA pool. The human ALDH1A3 cDNA was amplified by PCR from the human Mes GSCs cDNA pool using the following primer sequences: forward CACCGCCACCGCTAACGGGGCCGTG and reverse TCAGGGGTTCTTGTCGCCAAGTTTGATGGTGACAGT. The full-length ALDH1A3 open reading frame was cloned into the destination vector pDEST17 using pENTR/D-TOPO Invitrogen Gateway^®^ recombinant technology according to manufacture’s protocol. *E. coli* strain BL21(DE3) (Novagen) was transformed with the expression vector pDEST17-ALDH1A3 and was spread onto 2xTY agar plates with 50 μg/ml ampicillin for overnight growth at 37 °C. The next day, colonies were scraped and inoculated in 1 liter 2xTY medium supplemented with 50 μg/mL ampicillin. When a OD_600_ of 0.6–0.8 was reached, the temperature was shifted to 20 °C to induce the recombinant protein production. Induced cells collected by centrifugation, were re-suspended in 1/25 original volume of lysis buffer (50 mM Na_2_HPO_4_, 300 mM NaCl, 1mM β-mercaptoethanol, 10 mM imidazole at pH 7.5) and 250 units of benzonase nuclease. Plasma membranes of BL21(DE3) cells were fragmented by applying high pressure through the use of a French Press three times at 1.5 KBar. Protease inhibitor cocktail from SIGMA (100 μL per 40 mL lysis buffer) was added to the crude extract that was clarified by 1 h centrifugation at 18,000 rpm. The supernatant was purified by a His-tag affinity chromatography followed by size-exclusion chromatography, using an AKTA FPLC system at 4 °C. To evaluate the purity and homogeneity of the protein, after each purification step, eluted fractions were analyzed by SDS-PAGE and the protein quantification was always determined by Bradford protein assay. In the first purification step, the soluble fraction of recombinant human 6xHis-ALDH1A3 was purified with a Qiagen Ni-NTA Superflow 5 mL cartridge. The supernatant was loaded on the Ni-NTA column, previously equilibrated with 10 column volumes of lysis buffer. The Ni-NTA cartridge was washed with 50 mM Na_2_HPO_4_, 300 mM NaCl, 1 mM β-mercaptoethanol, 50 mM imidazole pH 7.5 until the absorbance at 280 nm return to the baseline (15 column volumes). The recombinant protein was eluted with 50 mM Na_2_HPO_4_, 300 mM NaCl, 1 mM β-mercaptoethanol, 250 mM imidazole pH 8 by applying a linear gradient in 20 column volumes. Eluted fractions were pooled and concentrated to 5 ml with Merck Millipore Amicon Ultra-15 10 kDa and loaded on a Sephacryl S200 16/60 column on AKTA FPLC system. Elution buffer contained 20 mM HEPES pH 7.5, 150 mM KCl, 1 mM β-mercaptoethanol, 0.5 mM EDTA and a flow rate of 0.2 mL/min was applied. This procedure allowed us to obtain 25 mg of pure and active ALDH1A3 used for all crystallization trials and kinetic analyses.

### Enzyme kinetic analysis and thermal shift assay

ALDH1A3 activity was followed by continuously measuring NADH formation at 340 nm (molar extinction coefficient of 6220 M^−1^ cm^−1^) for 30 minutes at 25 °C. The enzymatic assays were performed in a total volume of 200 μl in 20 mM Tris HCl pH 8.0, 1 mM β-mercaptoethanol, 150 mM KCl, with 2.6 μM of pure recombinant ALDH1A3. The kinetic parameters were determined by fitting the measured data to a Michaelis-Menten curve^41^ using SigmaPlot^42^. All the compounds were purchased from Sigma Aldrich and were dissolved in water except all-*trans* retinal that was dissolved in DMSO. For each assay the reaction mixture was pre-incubated for 1 minute in the absence of the enzyme and than the ALDH1A3 was added to initiate the reaction. The concentrations of NAD^+^, all-*trans* retinal and acetaldehyde when held constant, were 500 μM, 100 μM and 20 mM respectively. The concentration of NAD^+^ was varied from 0.02 μM to 100 μM when all-*trans* retinal was held constant and from 1 μM to 10 mM when using acetaldehyde as substrate and was kept constant; for all-*trans* retinal the explored range was from 0.02 μM to 60 μM and for acetaldehyde from 0.05 mM to 20 mM. Fluorescence for the thermal shift assay was measured in a 48-well plate using the FAM channel of a MiniOpticon™ Real-Time PCR Detection System (Bio-Rad). The melting temperature (T_m_) of ALDH1A3 protein alone and incubated with different ligands was recorded in triplicate and calculated as described by Matulis *et al.*^43^. The melting temperature (T_m_) was calculated as the inflection point of the sigmoidal melt curve. The protein was stored in a buffer containing 20 mM HEPES pH = 7.5, 150 mM KCl, 1 mM β-mercaptoethanol, 0.5 mM EDTA and diluted to a final concentration of 1 mg/mL. The enzyme was mixed with an appropriate amount of stock solutions of NAD^+^, NADH, all-*trans* retinal and retinoic acid to a final concentration of 1 mM and with the fluorescence probe SYPRO^®^ Orange (Sigma–Aldrich) used at 1/4000 dilution. Data were collected and analyzed using CFX Manager™ Software (Bio-Rad) and SigmaPlot^42^.

### Crystallization and Structure Determination

Crystals of ALDH1A3 were obtained by using the vapour-diffusion technique in sitting drop and applying a spare-matrix-based strategy with a crystallization robot (Oryx4, Douglas Instruments). The best crystals were grown by mixing 0.5 μl of protein solution at a concentration of 5.5 mg/mL, pre-incubated with 1 mM NAD^+^ and 1 mM REA, with an equal volume of a reservoir solution containing 20% PEG 3350, 0.24 M Na_2_ malonate pH 7.0, 10 mM TCEP hydrochloride and equilibrated against 50 μl of the reservoir solution, at 20 °C in about 30 days. Crystals of human aldehyde dehydrogenase 1A3 could only be obtained when both retinoic acid and NAD^+^ were present. For X-ray data collection, crystals were quickly equilibrated in a solution containing the crystallization buffer and 12.5% glycerol as cryo-protectant and flash frozen at 100 K in liquid nitrogen. Data up to 2.9 Å resolution were collected at the beamline ID23 EH1 of the European Synchrotron Radiation Facility (ESRF, Grenoble, France). Analysis of the diffraction data set allowed us to assign the crystal to the monoclinic P2_1_ space group with cell dimensions of a = 82.7 Å, b = 159.8 Å, c = 155.6 Å and β = 93.69°, containing eight molecules per asymmetric unit with a corresponding solvent content of 55.5%. Data were processed using the program package XDS^44^ and the CCP4 suite of programs^45^ was used for scaling. The structure determination of ALDH1A3 was carried out by means of the molecular replacement technique using the coordinates of the tetramer of *Ovis aries* ALDH1 as the search model (Protein Data Bank ID code 1BXS). PHASER^46^ was used to automatically determine the ALDH1A3 structure and returned a unique molecular replacement solution with an LLG of 4716.971 and TFZ = 11.3. The resulting electron density map was of high quality and allowed automatic tracing by the program AUTOBUILDING^47^. The initial model was subjected to iterative cycles of crystallographic refinement with the programs REFMAC5^48^ and PHENIX.REFINE^49^ using NCS restraints for all the 8 chains present in asymmetric unit, alternated with manual graphic session for model building using the program Coot^50^. 5% of randomly chosen reflections were excluded from refinement of the structure and used for the Free R factor calculation^51^. The program ARP/wARP^52^ was used for adding solvent molecules. NAD^+^ and REA were manually modelled based on both the 2*F*o − *F*c and *F*o − *F*c electron density maps (Supplementary Figure S4) with ideal geometry and an occupancy values for all the NAD^+^ and REA atoms of 1.0. Refinement was continued until convergence to R-factor and free R-factor values of 0.224 and 0.286 respectively, with ideal geometry. Data collection and refinement statistics are given in Table 1.

### Deposition

The atomic coordinates and structure factors of human ALDH1A3 have been deposited with the Protein Data Bank (www.rcsb.org) with the accession code 5FHZ.

### Illustrations

Figures were generated using the program pymol^53^.

## Additional Information

**How to cite this article**: Moretti, A. *et al.* Crystal structure of human aldehyde dehydrogenase 1A3 complexed with NAD^+^ and retinoic acid. *Sci. Rep.* **6**, 35710; doi: 10.1038/srep35710 (2016).

## Supplementary Material

Supplementary Information

## Figures and Tables

**Table 1:** Data collection and refinement statistics.

DATA COLLECTION	
Space group	P2_1_
*Cell dimensions*	
a, b, c (Å)	82.7, 159.8, 177.6
β(°)	93.7
Resolution (Å)	47.51 - 2.9
^R^_*pim*_/^R^_*merge*_	0.109(0.490)/0.184(0.785)
CC1/2	0.983 (0.534)
Mean(I)/sd(I)	5.4 (1.6)
Completeness (%)	98.3 (97.3)
Redundancy	3.6 (3.2)
**REFINEMENT**	
Resolution (Å)	2.9
Total reflections	364578 (31677)
Unique reflections	100139 (9865)
^R^_*work*_/^R^_*free*_	0.2244/0.2869
*No. atoms*	
Protein	29498
Water	63
Ligands	440
*Mean B-factors*	
Protein (Å^2^)	42.3
Water (Å^2^)	16.1
All Ligands (Å^2^)	48
REA_C (Å^2^)	26
NAD_C (Å^2^)	19
REA_D (Å^2^)	24
NAD_D (Å^2^)	28
*R.m.s deviations*	
Bond lengths (Å)	0.014
Bond angles (°)	1.48
*Ramachandran statistics*	
Residues in favoured region (%)	95.6
Residues in allowed region (%)	4.0
Residues in outlier region (%)	0.3
