# Supplementary material for: Crystal structure of human aldehyde dehydrogenase 1A3 complexed with NAD+ and retinoic acid
Source: Sci Rep. 2016 Oct 19;6:35710. doi: 10.1038/srep35710 (PMC5069622; doi:10.1038/srep35710)
Supplement: Supplementary Information [file srep35710-s1.pdf]

Crystal structure of human aldehyde dehydrogenase 1A3 complexed with NAD^+ and retinoic acid

Andrea Moretti[1], Jianfeng Li[2], Stefano Donini[1], Robert W. Sobol[2], Menico Rizzi[1*] and Silvia Garavaglia[1*].

[1]Department of Pharmaceutical Sciences, University of Piemonte Orientale, Largo Donegani 2, 28100 Novara, Italy; [2]Department of Oncologic Sciences, Mitchell Cancer Institute, University of South Alabama, Mobile, AL 36604, USA.

***Corresponding authors**

Silvia Garavaglia, [1]Department of Pharmaceutical Sciences, University of Piemonte Orientale, Via Bovio 6, 28100 Novara, Italy

Phone: +39 0321 375714

Fax: +39 0321 375821

Email: silvia.garavaglia@uniupo.it

Menico Rizzi, [1]Department of Pharmaceutical Sciences, University of Piemonte Orientale, Via Bovio 6, 28100 Novara, Italy

Phone: +39 0321 375712

Fax: +39 0321 375821

Email: menico.rizzi@uniupo.it

Keywords: Retinoic Acid, Aldehyde Dehydrogenase, ALDH1 family, NAD cofactor, Glioma/ Cancer Stem Cells.

Supplementary Table S1.

Steady-state kinetic parameters of hALDH1A3. The enzymatic assay has been conducted as described in the Experimental procedures. The reported values represent the average of three independent experiments.

Variable substrate	Fixed substrate				
		K_M	V_{max}	k_{cat}	k_{cat}/K_M
Acetaldhyde	NAD^+	2.4×10^{-3} M	5.5x10-5 μmol s^{-1}	0.1 s^{-1}	4.1×10^{1} $M^{-1}s^{-1}$
NAD^+	Acetaldehyde	77.5×10^{-6} M	7.6x10-5 μmol s^{-1}	0.1 s^{-1}	1.3×10^{3} $M^{-1}s^{-1}$
All-*trans* Retinal	NAD^+	9.3×10^{-6} M	1.1x10-3 μmol s^{-1}	1.6 s^{-1}	1.7×10^{6} $M^{-1}s^{-1}$
NAD^+	All-*trans* Retinal	4.8×10^{-6} M	1.4x10-3 μmol s^{-1}	2.0 s^{-1}	4.2×10^{6} $M^{-1}s^{-1}$

Supplementary Table S2.
Tm value for ALDH1A3 alone or in complex with oxidized and reduced cofactor and with all-*trans* retinal and retinoic acid. The assay has been performed as detailed in the Experimental procedures. An increased T_m indicates an augmented stability.

Protein	Ligand	Tm	ΔTm
Human ALDH1A3	/	57.5 °C	
	NAD^+	65.8 °C	8.3 °C
	NADH	59.8 °C	2.3 °C
	All-*trans* Retinal	58.3 °C	0.8 °C
	Retinoic Acid	60.0 °C	2.5 °C
	NAD^+ + All-*trans* Retinal	63.0 °C	5.5 °C
	NAD^+ + Retinoic Acid	64.5 °C	7.0 °C

Supplementary Table S3.
Residues omitted in the final model. List of the hALDH1A3 residues in the different eight molecules present in the asymmetric unit for which no electron density was visible in the refined structure.

**hALDH1A3**		***Missing residues***
Tetramer **ABCD**	Monomer **A**	1-18, 508-512
	Monomer **B**	1-22, 509-512
	Monomer **C**	1-20, 509-512
	Monomer **D**	1-19, 508-512
Tetramer **EFGH**	Monomer **E**	1-22, 508-512
	Monomer **F**	1-27, 338-343, 348-353, 386-394, 406-414 508-512
	Monomer **G**	1-28, 383-391, 404-411, 508-512
	Monomer **H**	1-20, 509-512

Supplementary Figure S4. Electron density maps around NAD^+ and REA in their respective binding sites. The Omit Fo–Fc map is shown in orange contoured at 2.5 standard deviations. The ligands NAD^+ and REA are shown as green and yellow sticks, respectively. The aminoacids defining the ligands pockets are represented as sticks. **A**) Electron density maps around NAD^+ and REA in complex with the monomer C in four different orientations spaced by a 90° rotation (0°, 90°, 180°, 270°). As the carboxyl terminal or REA_C is highly disordered the relative atoms -CH-C(CH3)-CH-COO- have been given zero occupancy in the coordinates file deposited with the protein Data Bank and are here reported in a conformation that is dictated by the stereo-chemical constrains given the preceding carbon-carbon double bond that is in the trans conformation being the ligand all-*trans* retinoic acid. **B**) Electron density maps around NAD^+ and REA in complex with the monomer D in four different orientations spaced by a 90° rotation (0°, 90°, 180°, 270°). The figure was prepared using the program PyMol **[53].**

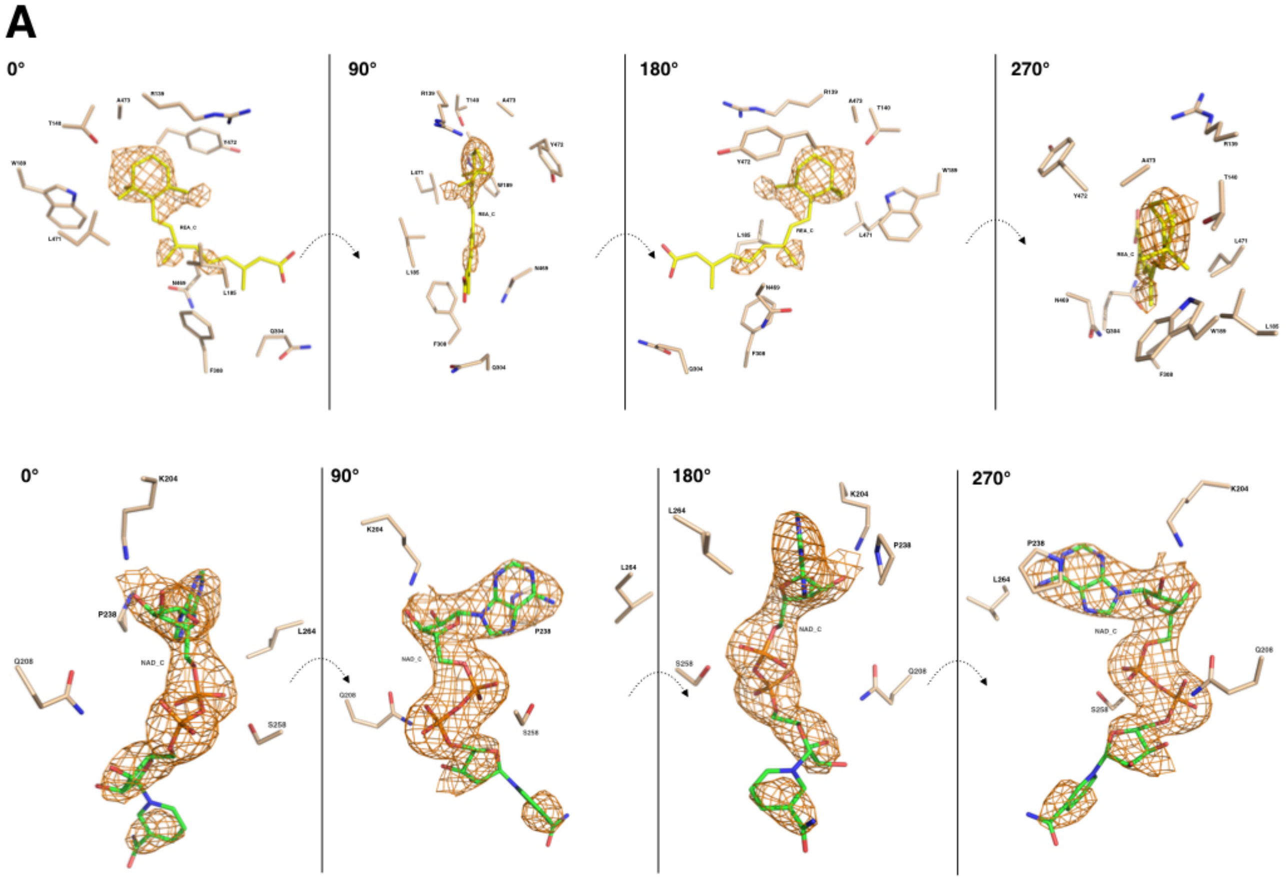

B

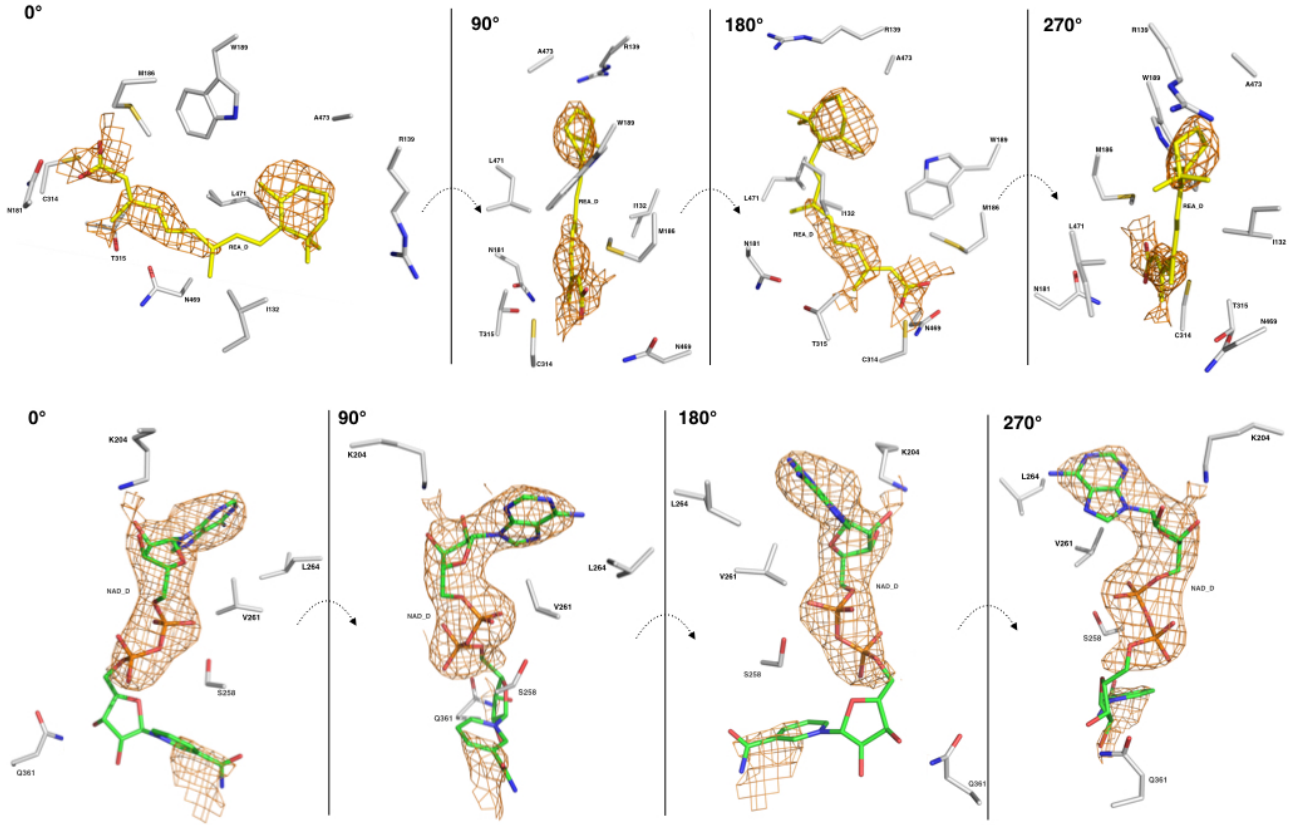

0°
90°
180°
270°
REA_D
NAD_D
W189
M186
A473
R139
N181
C314
L471
T315
N469
I132
K204
L264
V261
S258
Q361
